# Integrating Facility Metrics With Grassroots Insights: A Mixed-Methods Study to Improve Kayakalp Outcomes at Health and Wellness Centres in Ganjam, Odisha

**DOI:** 10.7759/cureus.90532

**Published:** 2025-08-19

**Authors:** Madhumita Bhakta, Durga M Satapathy, Jasmin N Panda, Pramila Marandi, Abhimanyu Behera, Anand K Sahu, Vidhyasaharan Rajagopalan

**Affiliations:** 1 Community Medicine, Maharaja Krushna Chandra Gajapati Medical College and Hospital, Berhampur, IND; 2 Community Medicine, Saheed Laxman Nayak Medical College and Hospital, Koraput, IND

**Keywords:** community health services, community participation, health and wellness centres, hygiene, kayakalp, quality improvement, sanitation

## Abstract

Background: The Kayakalp initiative by the Government of India aims to improve cleanliness, hygiene, and infection control across public health facilities, including health and wellness centres (HWCs). While facility-level assessments offer objective measurements, the lack of community perspectives limits the depth of quality evaluations.

Objectives: This study aimed to (i) evaluate the performance of HWCs using standard Kayakalp criteria, (ii) explore community healthcare workers’ perceptions regarding hygiene, service quality, and cleanliness, and (iii) develop community-driven recommendations for sustainable quality improvement.

Methodology: A mixed-method design was employed. The quantitative component involved a cross-sectional assessment of 36 selected HWCs using a structured Kayakalp checklist. Facilities were included based on operational status, infrastructure, manpower, and logistics. The qualitative component followed a phenomenological approach with purposive sampling. In-depth interviews (IDIs) and key informant interviews (KIIs) were conducted with frontline workers and analyzed through inductive content analysis.

Results: A total of 36 facility assessments revealed significant gaps in operational efficiency, manpower deployment, and adherence to hygiene protocols. Qualitative findings from 16 interviews highlighted frontline workers’ concerns about irregular cleaning staff, limited funding for sanitation, and challenges in community engagement. Thematic analysis identified key issues: manpower inadequacy, inaccessible facility locations, inconsistent waste management, and lack of community participation. A novel HYGIENE (Harness, Yield, Guide, Implement, Evaluate, Nurture, Enhance) model was proposed for improving Kayakalp compliance.

Conclusion: Integrating community insights with formal assessments exposed critical gaps in the implementation of Kayakalp at the grassroots level. Addressing these gaps requires policy-driven solutions, including dedicated funding, recruitment of cleaning staff, strategic relocation of HWCs, and community-led monitoring mechanisms. The study proposes scalable interventions to strengthen quality care and public satisfaction through enhanced hygiene practices and participatory governance.

## Introduction

India’s public healthcare system is undergoing a significant transformation with an emphasis on strengthening primary healthcare services through strategic policy initiatives. One of the most ambitious programs in this regard is the Ayushman Bharat initiative, launched by the Government of India in 2018, which aims to achieve universal health coverage (UHC) by transforming existing Sub Centres and Primary Health Centres into fully functional Health and Wellness Centres (HWCs). These centres are envisioned to provide comprehensive primary healthcare services, including maternal and child health, non-communicable disease management, and basic diagnostic and drug services, with a strong focus on community engagement and health promotion [1]. As a complementary effort to improve the quality of services provided at public health facilities, especially with regard to hygiene and infection control, the Ministry of Health and Family Welfare introduced the Kayakalp initiative in 2015. Kayakalp, meaning "transformation" in Sanskrit, is designed to promote cleanliness, hygiene, and infection control practices across government health facilities. The program recognizes and awards public health facilities that demonstrate high standards of cleanliness and adherence to infection control protocols [2]. The core philosophy of Kayakalp lies in instilling a culture of continuous quality improvement by conducting regular assessments and encouraging facilities to meet benchmark standards.

The assessment under Kayakalp is conducted using a structured checklist that evaluates facilities across thematic areas such as hospital upkeep, sanitation and hygiene, waste management, infection control, support services, hygiene promotion, and beyond the hospital boundary. These assessments are carried out both internally and externally, with performance scores determining the eligibility for awards and recognitions [3]. Over the past several years, the Kayakalp initiative has been successful in sensitizing healthcare providers to the importance of hygiene and has contributed to noticeable improvements in hospital environments, particularly in higher-tier facilities. Despite these achievements, a growing body of literature suggests that the current evaluation approach under Kayakalp remains largely facility-centric, emphasizing objective structural metrics while underrepresenting the lived experiences and perceptions of healthcare users and providers at the community level [4,5]. Numerous studies have documented tangible improvements in infrastructure maintenance, cleanliness protocols, and staff hygiene practices as a result of Kayakalp implementation, especially in district hospitals and urban health centres [6,7]. However, few studies have critically explored the challenges and perceptions of frontline workers in peripheral and rural HWCs, where resource constraints and logistical challenges are more pronounced [4,7].

Community health workers such as Accredited Social Health Activists (ASHAs), Auxiliary Nurse Midwives (ANMs), Community Health Officers (CHOs), and female multipurpose workers play a critical role in the day-to-day functioning of HWCs. These grassroots-level functionaries not only support clinical and outreach services but also act as liaisons between the health system and the community. Their perceptions, experiences, and challenges offer invaluable insights into the functional realities of HWCs, particularly concerning cleanliness, waste disposal, patient satisfaction, and community involvement [8]. Yet, their voices are often absent in formal assessments or quality improvement frameworks, limiting the depth and contextual relevance of the interventions proposed under programs like Kayakalp [7,8].

In rural and semi-urban regions, HWCs face several additional challenges that go beyond the standard Kayakalp checklist. These include geographic inaccessibility, temporary or contractual staffing, lack of essential supplies, intermittent electricity and water supply, and poor community ownership of health infrastructure. Such conditions hinder the consistent implementation of hygiene protocols and reduce the long-term sustainability of Kayakalp gains [9]. Moreover, the overemphasis on short-term compliance for assessment purposes, rather than long-term behavioral and systemic change, weakens the effectiveness of the initiative. In the absence of community-led monitoring and participatory governance, cleanliness often remains superficial and fails to create a culture of hygiene. The World Health Organization (WHO) emphasizes the critical importance of community participation in strengthening primary healthcare systems. According to WHO guidelines, sustainable improvements in hygiene and infection control can only be achieved when health systems are responsive to local contexts and involve the community in decision-making and monitoring processes [10]. However, this community-based participatory approach is not yet fully institutionalized in the implementation framework of Kayakalp, creating a disconnect between program goals and on-ground realities.

In light of these gaps, there is an urgent need to adopt a more integrative evaluation model that combines objective facility-level assessments with subjective insights from community stakeholders. Such a mixed-method approach not only helps identify the systemic bottlenecks in Kayakalp implementation but also highlights community-driven strategies that are contextually relevant and sustainable. Incorporating qualitative data from frontline health workers and community members can reveal hidden dimensions of quality, such as interpersonal relations, perceived cleanliness, usability of sanitation facilities, and the socio-cultural factors influencing hygiene behaviors.

Therefore, the present study was conceptualized to address the existing evaluation gap in Kayakalp implementation by blending quantitative facility assessments with qualitative community insights. By adopting a mixed-method research design, this study aims to capture both the measurable performance indicators and the experiential narratives of those who operate within and rely on HWCs. The ultimate goal is to propose actionable, evidence-based, and community-aligned recommendations to improve the quality of care and hygiene standards in HWCs in a sustainable and scalable manner. The specific objectives of this study were to evaluate the performance of selected HWCs based on Kayakalp criteria, to explore the perceptions of community-level healthcare workers regarding cleanliness, hygiene practices, and service quality at HWCs, and to develop actionable, community-led recommendations to strengthen the implementation and sustainability of Kayakalp at the primary healthcare level.

## Materials and methods

This study adopted a mixed-methods research design of explanatory sequential type, incorporating both quantitative and qualitative components to provide a comprehensive understanding of Kayakalp implementation at the HWC level, where qualitative exploration follows the quantitative assessments. The quantitative component was based on a cross-sectional facility assessment using a structured checklist, while the qualitative component followed a phenomenological approach to explore the lived experiences and perceptions of community health workers. The study was conducted in selected HWCs under the jurisdiction of a rural and semi-urban block within Ganjam district, Odisha, India. These HWCs function under the National Health Mission and represent varying levels of infrastructure, manpower availability, and community engagement. The study was carried out over a period of 12 months, from January 2024 to January 2025. This included preparatory activities such as tool development and pilot testing, data collection (both quantitative and qualitative), and analysis.

For the quantitative component, the study population included operational HWCs that had not previously been certified under the National Quality Assurance Standards (NQAS). For the qualitative component, the study population comprised community-level healthcare providers, including CHOs, ASHAs, female multipurpose health workers, and other frontline workers directly involved in the functioning of HWCs. A multistage sampling method was used for selecting HWCs in the quantitative phase. In the first stage, six blocks within the district were randomly selected out of the 22 blocks. In the second stage, six HWCs within each selected block were purposively chosen based on their operational status and diversity in geography and accessibility. For the qualitative phase, purposive sampling was used to select participants for in-depth interviews (IDIs), ensuring representation from various cadres of community health workers. A total of 36 HWCs were assessed using the Kayakalp checklist. For the qualitative component, 16 IDIs were conducted, reaching data saturation. HWCs that were functional and operational during the study period, community health workers with at least one year of experience in their current role and participants who provided informed consent to participate in the study were included in the study while HWCs that were undergoing major renovations or logistical disruptions during the study period, and facilities already certified under NQAS were excluded.

The study was implemented in two sequential but integrated phases. In the first phase, field visits were conducted to selected HWCs to assess their performance using the Kayakalp assessment checklist. Each facility was evaluated across eight thematic areas. In the second phase, qualitative interviews were conducted with community health workers using a semi-structured interview guide to explore perceptions, barriers, and enablers related to hygiene practices and Kayakalp implementation. For the quantitative phase, a structured Kayakalp facility assessment checklist developed by the Ministry of Health and Family Welfare (MoHFW) was used (see Appendix A). It covered core domains. For the qualitative phase, a semi-structured interview guide was developed based on a literature review and expert input (see Appendix B). The tool included open-ended questions to capture experiences, perceived challenges, and suggestions for improving hygiene and quality of care at HWCs. Quantitative data were collected by trained investigators through direct observation, record review, and interaction with facility staff. Each assessment took approximately two to three hours per facility. Qualitative data were collected through face-to-face in-depth interviews conducted at participants’ workplaces or a neutral location. Interviews were conducted in the local language (Odia), audio-recorded with consent, and supplemented with field notes. Each interview lasted for about 15-30 minutes. Data collection continued until thematic saturation was achieved.

The primary outcome for the quantitative component was the Kayakalp performance score across various domains. Secondary outcomes included identification of domain-specific gaps and deviations from protocol. In the qualitative component, the primary outcomes were the emergent themes and subthemes related to the perceived implementation challenges and opportunities in maintaining cleanliness, hygiene, and infection control practices at HWCs. Quantitative data were entered into Microsoft Excel (Microsoft Corporation, Redmond, Washington, United States) and analyzed using descriptive statistics such as means, medians, and domain-wise scoring percentages. Qualitative data were transcribed verbatim, translated into English, and analyzed using manual content analysis. An inductive coding approach was used, with codes grouped into themes and subthemes using thematic analysis. A code cloud was generated to visualize the frequency of emerging codes. Rigorous quality control measures were adopted at each stage. All tools were pre-tested in a non-study HWC to ensure contextual relevance. Field investigators were trained thoroughly to ensure standardization. For qualitative interviews, data triangulation was ensured by comparing findings from IDIs. Member checking and peer debriefing were conducted to enhance the credibility and trustworthiness of qualitative findings.

The study protocol was reviewed and approved by the Institutional Ethics Committee of Maharaja Krushna Chandra Gajapati Medical College and Hospital, Berhampur, Odisha (approval number: 06, dated August 21, 2024). Written informed consent was obtained from all participants prior to data collection. Participants were assured of confidentiality and the right to withdraw at any time without consequences. Data were anonymized during transcription and analysis. Findings were disseminated to stakeholders for policy advocacy and program improvement in line with ethical obligations.

## Results

Quantitative assessment

**Figure 1:**
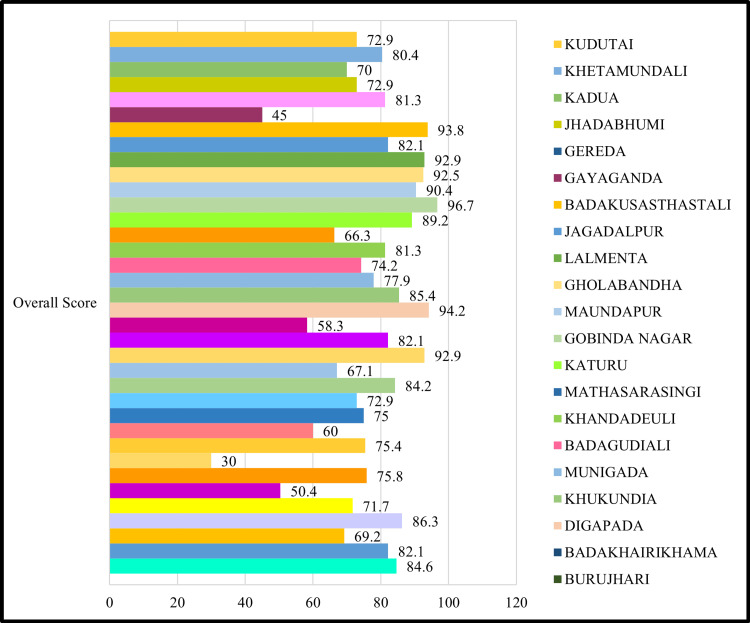
Kayakalp domain-wise scores across 36 health and wellness centres in Ganjam district, Odisha Each colored bar in the figure represents the performance score of an individual Health and Wellness Centre (HWC) based on the Kayakalp assessment

A total of 36 HWCs across Ganjam district were assessed using the standard Kayakalp checklist (Figure 1). Among these, 26 facilities (72%) achieved an overall score above the 70% threshold, indicating satisfactory compliance with Kayakalp standards. Some facilities, 10 HWCs (28%), scored below the benchmark, suggesting significant gaps in quality assurance and hygiene practices.

**Figure 2:**
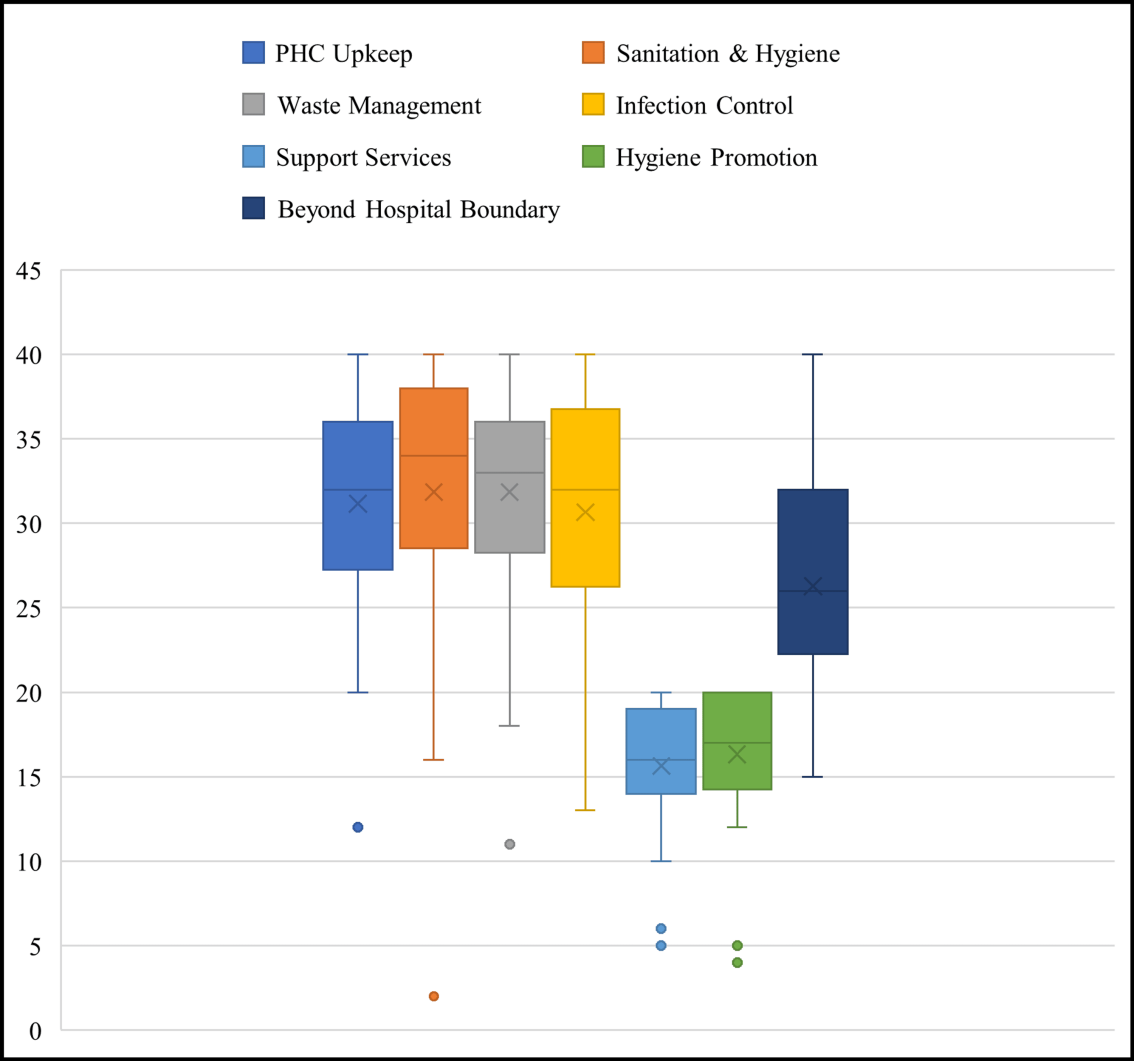
Domain-wise distribution of Kayakalp scores across health and wellness centres in Ganjam district (N = 36) This box-and-whisker plot illustrates the distribution of Kayakalp performance scores across five key thematic domains evaluated at 36 Health and Wellness Centres (HWCs) in Ganjam district. Each colored box represents a specific domain: hospital upkeep (blue), sanitation and hygiene (orange), biomedical waste management (grey), infection control (yellow), and support services (green). The central line within each box indicates the median score, while the "X" symbol denotes the mean. The length of the box shows the interquartile range (IQR), capturing the middle 50% of scores. Whiskers extend to the minimum and maximum values within 1.5 times the IQR, and any data points beyond this range are represented as individual outliers (dots)

Performance across the seven thematic domains varied considerably, as seen in Figure 2. In the domain of hospital upkeep, 22 HWCs (61%) scored above 60%, while 14 HWCs (39%) fell below this threshold. The average score in this domain was 62%, reflecting moderate adherence to structural cleanliness and maintenance protocols. In the domain of sanitation and hygiene, only 17 facilities (47%) attained scores above 60%, whereas 19 HWCs (53%) were below the acceptable mark. The average score for this domain was 58%, indicating that daily cleaning routines and hygiene materials were inconsistently maintained. Biomedical waste management emerged as a critical deficiency area. Only 11 HWCs (31%) scored above 60%, while the remaining 25 facilities (69%) had significant lapses in segregation, disposal, or availability of functional equipment. The mean score in this domain was 49%. Similarly, infection control practices were found to be inadequate in 21 HWCs (58%), with only 15 facilities (42%) meeting minimum compliance levels. The average domain score was 52%, pointing to gaps in the use of personal protective equipment (PPE), surface disinfection, and training. The most poorly performing domain was support services, with just eight HWCs (22%) scoring above 60%, while 28 HWCs (78%) performed poorly. The average score in this category was only 45%, reflecting systemic deficiencies in pest control, water supply, lighting, and auxiliary manpower. Additional facility-level observations corroborated these domain-wise findings. Notably, 26 HWCs (72%) reported the absence of designated cleaning or sanitation staff. In these facilities, cleaning duties were informally undertaken by multipurpose health workers, ANMs, or even patient attendants, often without appropriate protective gear or supervision. Furthermore, 22 HWCs (61%) lacked functional biomedical waste disposal infrastructure, including color-coded bins and incinerators, which compromised safe waste handling. Water availability also emerged as a key challenge, with 14 HWCs (38%) reporting irregular or no piped water supply, directly impacting hand hygiene and surface cleaning. Additionally, nine HWCs (25%) were located outside the core village settlement, on the periphery, or in isolated areas. Such locations contributed to reduced accessibility, lower community footfall, and weaker community engagement in cleanliness efforts.

Qualitative assessment

The quantitative assessment highlights substantial variability in Kayakalp performance across HWCs, with consistent underperformance in key areas such as waste management, infection control, and infrastructure support. These findings suggest the need for targeted interventions to improve both the structural and operational aspects of primary health facility hygiene and service delivery. These numbers, though informative, only scratched the surface. To move beyond the scores and understand the “why” behind these performance gaps, a qualitative exploration was undertaken through in-depth and key informant interviews. These included ASHAs, female multi-purpose health workers, CHOs, and male multipurpose workers, individuals deeply embedded in the day-to-day realities of HWCs. Their stories, emotions, and frustrations went far beyond the static checklist scores of Kayakalp assessments. When analyzed through an inductive thematic lens, their voices coalesced into six core themes that bring to life the challenges and opportunities at the frontline of hygiene and quality care.

**Figure 3:**
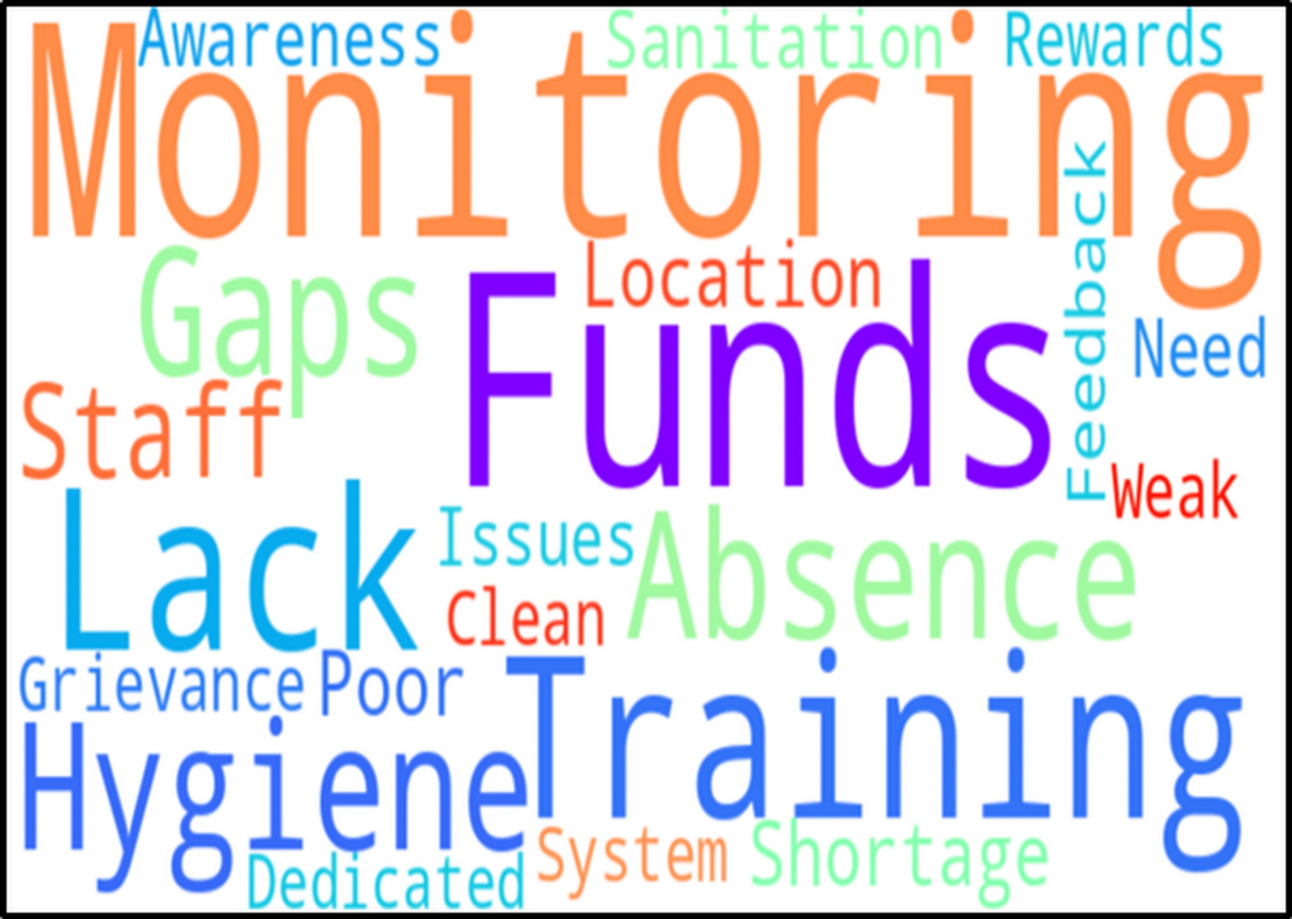
Code cloud reflecting frontline perspectives on barriers and enablers in Kayakalp implementation at health and wellness centres This code cloud visually represents the most frequently recurring codes. The font size indicates the relative frequency and emphasis placed on that code during interviews, and the color variation enhances visual categorization without hierarchical bias. Larger and bolder words indicate issues that were repeatedly stressed, such as monitoring, funds, training, lack, absence, and hygiene

As the voices of frontline healthcare workers were transcribed and thematically coded, a clear and compelling picture began to emerge, a picture now encapsulated in a code cloud (Figure 3).

At the center of their concerns looms the word “Monitoring”, the most frequently cited issue. This isn't just about oversight; it speaks of a deeper absence of follow-through. Many workers described how the pressure to meet standards peaks during assessment periods, but quickly dissipates afterward. The lack of post-assessment monitoring leaves facilities in limbo, where standards fall as quickly as they rise. Close behind is “Funds”, a persistent plea across all interviews. Whether it was for cleaning supplies, equipment repair, or hiring sanitation staff, the absence of dedicated funding underpinned many of the program’s shortcomings. As one participant noted, “We want to do more, but we can’t even buy disinfectant without waiting for months.” “Training” also stands tall in the cloud. Workers frequently described being ill-prepared to meet Kayakalp standards, particularly in areas like biomedical waste segregation and infection control. The desire to learn and improve was palpable, but without systematic capacity-building, efforts remain inconsistent and vulnerable. Words like “Hygiene”, “Staff”, “Gaps”, and “Absence” cluster around the center, reflecting a broader concern with operational functionality. Facilities often lacked the manpower and routines needed to sustain basic hygiene practices. One multipurpose health worker admitted, “We do what we can, but there’s no one assigned to keep the place clean; it depends on who’s free that day.” The code “Lack” itself appears prominently, echoing across multiple subthemes. Lack of staff. Lack of supplies. Lack of awareness. Lack of coordination. It’s a haunting refrain that reverberates through the narrative of each facility. Also of note are codes such as “Awareness”, “Sanitation”, “Grievance”, and “System”, signaling unmet needs in community education, feedback mechanisms, and systemic accountability. When staff feel unheard, unsupported, and invisible, motivation wanes and performance suffers. Finally, some smaller, but significant, codes hint at possible solutions. “Feedback”, “Rewards”, and “Dedicated” appear like green shoots in an otherwise dry landscape, reminding us that improvement is possible where there is recognition, targeted funding, and consistent involvement.

**Figure 4:**
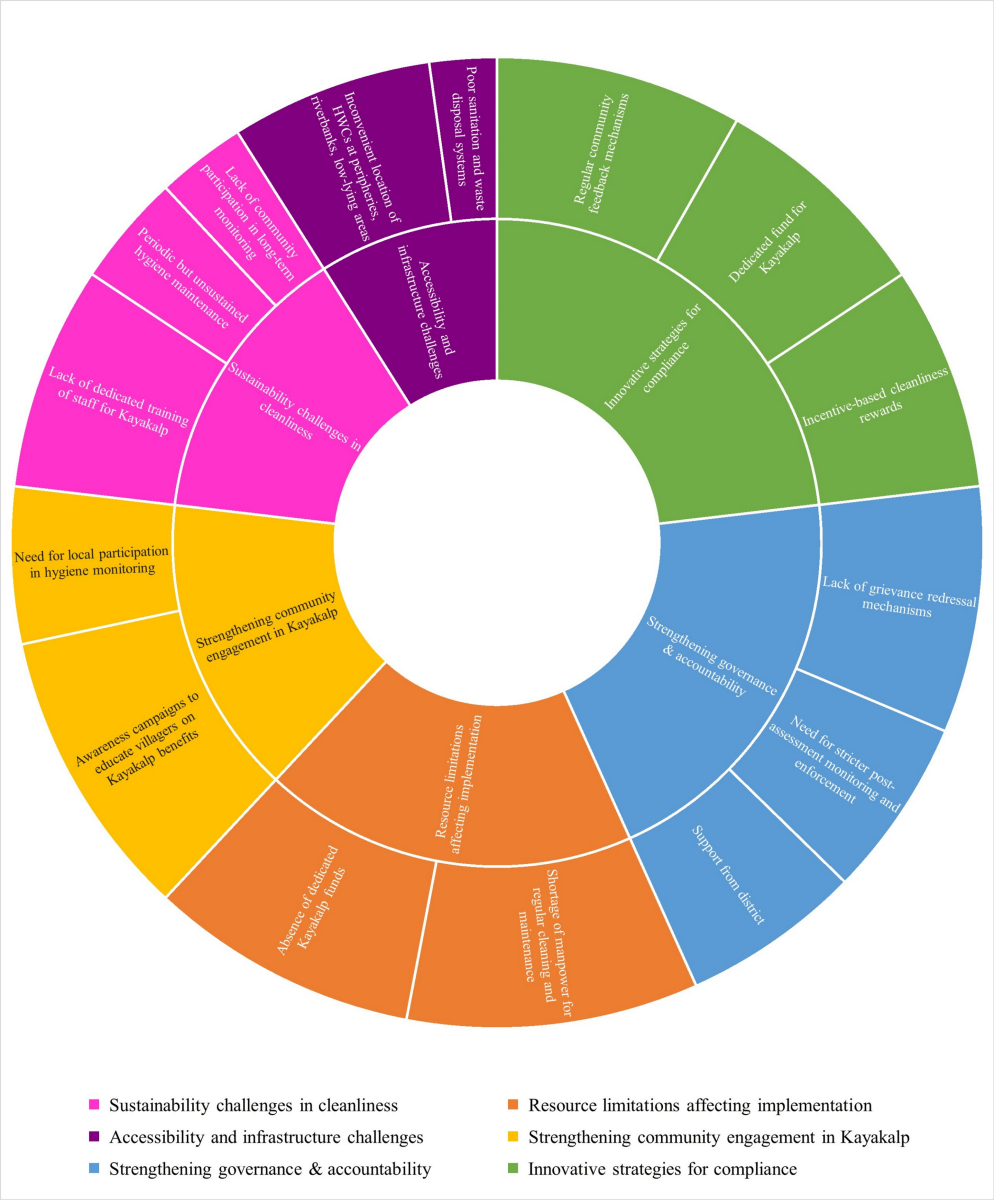
Thematic map of frontline health worker perspectives on Kayakalp Implementation in health and wellness centres, Ganjam District This sunburst diagram illustrates the six major themes that emerged from the qualitative interviews with 16 frontline healthcare workers. Each color-coded segment represents a primary theme, and the outward branches depict the sub-themes or specific issues raised within that category

The thematic sunburst image (Figure 4) visualizes the six key themes that emerged from this qualitative inquiry, each theme radiating outward to reveal specific sub-themes or grounded issues identified by participants. It serves as a conceptual bridge between the hard data and the nuanced, real-world struggles and strategies that define the day-to-day reality of Kayakalp implementation.

Theme 1: Sustainability Challenges in Cleanliness: a Short-Lived Shine

At the heart of this thematic framework is a systemic concern: cleanliness in HWCs is largely unsustainable without deeper structural and behavioral change. Participants described a recurring pattern of intense preparation for Kayakalp inspections, followed by a quick reversion to pre-assessment norms-often due to the lack of dedicated sanitation workers or the absence of supervisory oversight.

A recurring sentiment among participants was the impermanence of hygiene efforts-what some described as “event-based cleanliness.” The flurry of activity that surrounds Kayakalp inspections quickly fades once the assessors leave.

"Just before inspection, we are told to clean everything and arrange files. But after that, there is no follow-up. It doesn’t continue.”

~ Participant 6

“We used to clean with whatever was available: detergent powder and a mug. There is no permanent separate sanitation staff in our centre.”

~ Participant 13

These testimonies reveal how cleanliness is often reactive, driven more by deadlines than by institutional culture. The absence of routine supervision, structured cleaning schedules, and performance-linked incentives makes hygiene unsustainable. What emerges is a cycle of temporary compliance, quickly reverting to neglect in the absence of systemic reinforcement.

Theme 2: Resource Limitations: Managing with What Little There Is

Moving outward in the visual, the orange segments reveal how resource scarcity undermines even the most motivated efforts. In many HWCs, basic materials like cleaning agents and color-coded bins were unavailable, while biomedical waste was burned in open pits due to a lack of disposal systems. Staff shortages added another layer of burden, with multipurpose workers taking on janitorial roles in addition to clinical duties.

As we delved deeper, a picture of resource strain became apparent, one marked by missing staff, broken tools, and improvised solutions. Nearly every respondent pointed to the absence of designated cleaning personnel as a major operational gap. Infrastructure, too, seemed to crumble under pressure. Biomedical waste management was often compromised, with broken bins, missing lids, and informal burning practices. These situations not only violate infection control norms but also place undue burden on sanitation workers.

“The yellow and red bins are there, but the waste is thrown in a single bin.”

*~*Participant 3

Theme 3: Accessibility and Infrastructure: The Burden of Being Out of Sight

The purple segments speak about the physical and social inaccessibility of some facilities, for example, HWCs built on burial grounds or at village peripheries, deterring community members and leaving staff feeling unsafe and isolated. These were not just logistical issues but were deeply tied to how the community perceives and engages with these facilities.

In some parts of Ganjam, the HWCs themselves seemed forgotten, either by design or by neglect. Several facilities were located on the fringes of villages, disconnected from the communities they were meant to serve.

“Our HWC is built on a burial ground. No one wants to come here. Even we feel scared after dark.”

~ Participant 5

Physical remoteness further complicated matters.

“It is 3 kilometers outside the village; there’s no transport. Elderly people and women hesitate to walk here alone, especially during evening hours.”

~ Participant 2

The challenges extended to personal safety as well.

“There’s no guard posted here even at night. We do not feel safe staying late at night.”

*~ *Participant 2

Inaccessible locations, coupled with inadequate lighting and security, not only deterred patients but also left staff feeling vulnerable and unsupported.

Theme 4: Community Engagement: A Missing Pillar

The yellow sections point to the underutilized potential of the community in Kayakalp. Many health workers lamented that the initiative remained “a government thing” in the eyes of the villagers. Yet in places where self-help group (SHG) or Panchayat members were actively involved, hygiene was seen as a shared responsibility rather than an imposed obligation.

Despite being a program meant to celebrate cleanliness and community pride, Kayakalp seemed distant from the people it aimed to serve. Several respondents noted that community awareness was low, and engagement mechanisms were either absent or ineffective.

“Villagers don’t even know what Kayakalp is. For them, it’s just another government thing.”

*~ *Participant 14

Yet, where community institutions were involved, results seemed more promising.

“If the SHG women or panchayat members were involved, it would help maintain cleanliness and solve local issues faster.”

*~ *Participant 1

These reflections underscore the unrealized potential of community-led accountability and suggest that strengthening local participation could serve as a catalyst for sustained hygiene.

Theme 5: Governance and Accountability: A System Without Watchers

Governance issues are reflected in the blue wedges, which reveal a culture of weak accountability. Participants noted that recordkeeping had become symbolic; registers were filled out for compliance, but rarely reviewed. The need for independent audits, surprise checks, and grievance mechanisms was voiced repeatedly.

A critical insight emerging from the narratives was the weakness of governance mechanisms. Facility registers were being filled-sometimes diligently, but rarely reviewed. Supervisory visits from Medical Officers in Charge (MOICs) were infrequent and largely symbolic.

“Our MOIC comes once a month. There’s no one to check if bins are used or not.”

“We fill registers just for the sake of showing.”

This lack of oversight allows standards to erode silently. Staff, in turn, feel disconnected from institutional goals, leading to a breakdown in accountability and a widening gap between policy and practice.

Theme 6: Innovative Strategies: Hope in the Margins

The green segments showcase a spirit of innovation and resilience. From makeshift handwashing stations built with repurposed drums to wall murals created by school children, these micro-innovations were signs of ownership and agency at the ground level; examples of how compliance could be both cost-effective and community-driven.

Amidst these challenges, sparks of initiatives flickered across several HWCs. When resources failed, creativity often stepped in.

“We can hold hygiene sessions in the local school with support from the headmaster. Five students can be selected as 'Swachhata Monitors'. They will check water pots, cleanliness of the anganwadi, and even guide their families on handwashing and waste disposal.”

*~ *Participant 15

“At the end of each month, we can invite local SHG leaders, ward members, and youths to take a hygiene oath. Everyone will pledge to maintain cleanliness in their area. It will build a sense of unity and seriousness.”

*~ *Participant 10

“During every gram sabha, we can display photos of how the HWC looked before our cleaning efforts and how it looks now. It will make the community proud and also make people come to our HWC.”

*~ *Participant 16

These grassroots ideas were not only practical but deeply symbolic. They reflected ownership, resilience, and a desire to bridge systemic gaps with locally driven solutions. They serve as a reminder that when empowered, even the most resource-strapped facilities can generate sustainable, low-cost interventions with high impact.

Meta-inferencing matrix

To integrate the quantitative findings with the lived experiences of frontline workers, a meta-inferencing matrix (Table 1) was developed. This synthesis not only validated the performance gaps observed through Kayakalp assessments but also contextualized them within the operational realities and perceptions on the ground.

**Table 1:** Meta-inferencing matrix of key themes from mixed-method analysis The Meta-Inferencing Matrix integrates quantitative findings (such as Kayakalp scores and infrastructure statistics) with qualitative insights (like field observations and stakeholder interviews) to draw comprehensive inferences on key themes affecting cleanliness and hygiene in healthcare settings. Each theme—ranging from sustainability challenges and resource limitations to governance gaps and innovative practices—is examined through both data strands. Quantitative evidence highlights measurable deficiencies, while qualitative insights reveal ground-level experiences and contextual challenges. The integrated inference column synthesizes these strands to offer a holistic understanding of barriers and opportunities for effective and sustained hygiene practices. HWC: health and wellness center; MOIC: medical officer in charge; SHG:

Theme	Quantitative Evidence	Qualitative Insight	Integrated Interface
Sustainability Challenges in Cleanliness	<60% scores in Sanitation & Infection Control domains	Hygiene improvements limited to assessment periods	Lack of routine monitoring and sustained motivation impedes long-term cleanliness culture
Resource Limitations	72% lacked cleaning staff; 61% lacked proper waste management setup	Staff and patients forced to manage cleaning; broken equipment common	Operational and material constraints severely limit effective Kayakalp implementation
Accessibility & Infrastructure	25% HWCs located outside village boundaries	HWCs built on burial grounds or remote sites; safety issues prevalent	Inaccessibility and unsafe infrastructure reduce usage and compromise hygiene maintenance
Community Engagement	Limited community-based feedback systems	Panchayats and SHGs not involved; low public awareness	Low community ownership hinders accountability and behavioral change
Governance & Accountability	Weak scores in Support Services and recordkeeping	Irregular MOIC supervision; symbolic documentation	Governance failure allows hygiene compliance to decline post-assessment
Innovative Strategies for Compliance	Not measured in Kayakalp scores	Improvised handwashing units and creative community engagement practices observed	Low-cost, grassroots innovations offer scalable solutions for hygiene improvement

Table 1 clearly illustrates that underperformance in domains such as sanitation, infection control, and support services is deeply rooted in systemic barriers, ranging from resource shortages and inadequate infrastructure to weak accountability and limited community engagement. Quantitative scores alone could not explain why some facilities failed to sustain hygiene practices; however, qualitative insights revealed the central role of motivation, supervision, and ownership in maintaining compliance beyond inspections. Importantly, while governance failures and resource deficits emerged as dominant barriers, the matrix also highlighted the promising role of grassroots innovation, unmeasured in routine assessments but richly described in interviews as a potential pathway toward sustainable and scalable improvement.

## Discussion

The present study employed a mixed-method approach to assess the implementation of the Kayakalp program in 36 HWCs across Ganjam district. While quantitative assessments using the standard Kayakalp checklist revealed widespread underperformance across key hygiene and infection control domains, qualitative insights uncovered systemic, contextual, and operational barriers that explain the persistent quality gaps at the primary care level.

Only 10 of the 36 HWCs (28%) assessed did not score above the 70% compliance threshold, indicating that the majority of facilities (72%) were able to meet basic standards of hygiene and infection control. These findings are consistent with previous state-level evaluations that highlight variable and often suboptimal implementation of Kayakalp, particularly in peripheral health facilities [6]. Domain-wise analysis further illustrated this gap: while hospital upkeep showed moderate adherence (mean score 62%), critical domains such as biomedical waste management (49%), infection control (52%), and support services (45%) remained significantly deficient. These results echo similar studies from other states that reported systemic challenges in maintaining infection control and sanitation due to infrastructural and human resource constraints [11,12]. The most prominent structural gap was the widespread absence of dedicated sanitation staff in 72% of the facilities, which directly impacted the consistency of cleaning routines. In the absence of institutionalized staff support, frontline health workers were forced to take on janitorial responsibilities, often informally and without protective equipment. Additionally, 61% of facilities lacked proper biomedical waste disposal systems, and 38% faced unreliable water supply, factors that severely compromised both hygiene and infection control. These findings align with prior research highlighting that quality deficits in rural health centres often stem from fragmented supply chains, irregular resource flow, and minimal infrastructural investment [9]. Perhaps more concerning was the revelation that 25% of the HWCs were located outside the main village settlement, some on socially stigmatized sites such as burial grounds, raising serious concerns about accessibility, community trust, and safety. This spatial disconnect not only hindered service utilization but also discouraged ownership and accountability from both staff and community. Such infrastructural and locational barriers have previously been noted as critical deterrents to effective primary healthcare utilization and community engagement [10].

The qualitative component of the study significantly enriched these findings. In-depth interviews with ASHAs, ANMs, CHOs, and multipurpose health workers revealed that hygiene activities were often reactive and inspection-driven, characterized by a visible spike in efforts just prior to assessments, followed by a swift decline. This pattern, described by participants as “event-based cleanliness,” underscores the program’s current dependency on external monitoring rather than internalized quality culture. These observations align with existing critiques that, while Kayakalp has improved awareness and documentation, it has yet to fully embed sustained behavioral change at the facility level [13,14].

A particularly revealing dimension of the study was the role of governance. Participants frequently cited symbolic documentation and irregular supervisory visits from MOICs, which contributed to a lack of accountability. Recordkeeping was perceived more as a bureaucratic formality than a tool for quality improvement. This aligns with findings from other studies that emphasize the need for robust internal audits and real-time feedback systems to sustain improvements in healthcare quality [6,15]. Community engagement emerged as another crucial gap. Several participants noted that villagers were largely unaware of the Kayakalp program and did not see it as a community-owned initiative. However, in facilities where local institutions such as SHGs and Panchayats were actively involved, hygiene practices were reported to be more consistent and resilient. These findings support the WHO’s assertion that community participation is integral to primary health system strengthening [10] and suggest that the inclusion of local governance bodies in Kayakalp monitoring could improve both compliance and ownership.

Despite the multiple challenges, the study also highlighted areas of promise. Innovative strategies, such as constructing handwashing stations from recycled materials or mobilizing schoolchildren for hygiene-related wall art, demonstrated the potential for low-cost, high-impact solutions. These grassroots innovations, though unmeasured in formal assessments, represent the creative agency of frontline workers in the face of resource limitations. Recognizing and scaling such initiatives could serve as a powerful lever for reform. The meta-inferencing matrix developed in this study helped bridge the quantitative and qualitative findings. It showed that low scores in key domains were not isolated issues but reflected deeper systemic constraints, such as staff shortages, lack of supervision, and minimal community involvement. Importantly, the matrix also revealed that while the Kayakalp checklist captures surface-level hygiene indicators, it fails to account for contextual enablers such as innovation, motivation, and social participation. Collectively, these insights underscore the need to transition Kayakalp from a compliance-based evaluation model to a more holistic, community-anchored framework. Programmatic improvements should include the appointment of dedicated sanitation staff, reliable funding streams for hygiene supplies, structured capacity-building programs, and formal mechanisms for community monitoring and feedback. Strengthening governance through routine supervision, transparent grievance redressal, and incentivized performance can further reinforce accountability.

**Figure 5:**
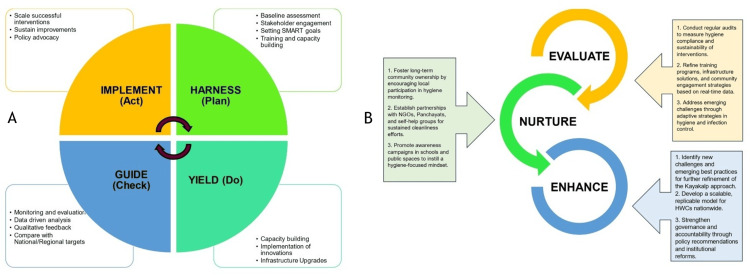
HYGIENE (Harness, Yield, Guide, Implement, Evaluate, Nurture, Enhance) model for enhancing Kayakalp at health and wellness centres Image Credit: Authors

Building on the findings of this study and grounded in real-world operational insights, a cyclical and adaptive framework is proposed to transform the implementation of Kayakalp in primary healthcare settings (Figure 5). This framework recognizes that maintaining hygiene and quality standards is not a one-time effort but a continuous, evolving process requiring coordinated planning, execution, feedback, and system-wide adaptation.

Figure 1A shows the classic Plan-Do-Check-Act (PDCA) cycle in the context of HWCs. It begins with HARNESSING baseline data through community mapping, stakeholder consultations, and training needs assessment. Here, the setting of SMART goals (Specific, Measurable, Achievable, Relevant, Time-bound) ensures targeted and trackable actions. This feeds into the YIELD phase, where ideas are translated into action through capacity-building workshops, infrastructure upgrades, and pilot implementation of innovative solutions-such as locally developed handwashing stations or awareness campaigns led by school children. As these interventions are deployed, the system must then GUIDE the process through monitoring and evaluation. This includes regular supervisory visits, use of standard checklists, and capturing of qualitative feedback from both health workers and community members. Crucially, this phase allows the identification of gaps and successes relative to national benchmarks, promoting evidence-based refinements. Finally, the IMPLEMENT phase acts on these insights to scale up what works and discontinue what doesn't-embedding policy changes, allocating dedicated funds, and sustaining momentum through advocacy. Parallelly, the spiral model on the right (Figure 1B) proposes an iterative, forward-moving process of Evaluate, Nurture, and Enhance (ENE). The Evaluate stage involves regular audits and root cause analysis of hygiene compliance gaps, particularly in domains like biomedical waste management and infection control. The Nurture stage emphasizes fostering long-term community ownership, encouraging SHGs, Panchayats, and local schools to participate actively in hygiene maintenance and behavior change campaigns. This creates a culture of shared responsibility, as opposed to top-down compliance. The final phase, Enhance, focuses on institutionalizing change. This includes identifying replicable models from high-performing HWCs, integrating them into broader district health strategies, and strengthening governance mechanisms. Recommendations generated through this phase-such as dedicated sanitation staffing norms or district-level incentive schemes-can be routed into policy reforms for broader impact. This dual-model framework-one cyclical, one progressive-captures both the operational discipline and adaptive flexibility required to truly transform hygiene practices in HWCs. It aligns with the World Health Organization’s advocacy for continuous quality improvement embedded in primary healthcare systems [10]. 

Strengths and limitations

One of the major strengths of this study lies in its robust mixed-methods design, which effectively blended quantitative facility-based assessments with rich qualitative insights from frontline healthcare workers. This dual approach enabled the identification of not only the measurable gaps in Kayakalp performance but also the underlying systemic and contextual factors that contribute to these shortcomings. By capturing the authentic voices of ASHAs, ANMs, and CHOs, the study adds depth and credibility to the findings, ensuring that recommendations are grounded in real-world operational challenges. Another strength is the development of the innovative HYGIENE model, which translates these findings into a practical, cyclical roadmap for sustainable quality improvement in hygiene practices at HWCs. The study’s clear focus on community participation, frontline innovation, and governance accountability makes its insights highly relevant for policymakers and program managers aiming to strengthen primary healthcare delivery.

Despite its strengths, the study has certain limitations that should be acknowledged. Firstly, the cross-sectional nature of the quantitative assessment provides only a snapshot of facility performance, which may not fully capture seasonal or periodic variations in hygiene practices. The relatively small sample size of 36 HWCs and 16 qualitative interviews, while sufficient for thematic saturation, may limit the generalizability of findings beyond the study district. Social desirability bias may have influenced participants’ responses during interviews, potentially underreporting certain challenges or overstating compliance efforts. Moreover, the study did not include direct perspectives from patients or community members, which could have provided an additional dimension to the understanding of hygiene perceptions and expectations at the grassroots level. Finally, resource and time constraints restricted longitudinal follow-up, which would have been valuable to assess the impact of recommended interventions over time.

## Conclusions

The evidence presented in this study calls for a strategic shift in how the Kayakalp initiative is implemented and monitored at the grassroots level. Moving beyond compliance-driven audits, the integration of participatory planning, localized innovation, robust supervision, and institutional support is critical to achieve sustainable improvements. The dual-model framework presented, rooted in both the PDCA cycle and ENE spiral, offers a replicable and scalable roadmap for state and national program managers aiming to embed hygiene as a culture rather than a checklist. This integrated approach ensures that cleanliness becomes an everyday ethic, driven not only by assessments but by empowered staff, informed communities, and responsive systems. This study underscores the need for a paradigm shift in how the Kayakalp initiative is implemented and sustained at the primary healthcare level. By revealing the complex interplay of structural inadequacies, governance gaps, limited community engagement, and the creative resilience of frontline workers, the findings highlight that true improvement in hygiene standards requires more than compliance checklists. The proposed HYGIENE model offers a practical, cyclical framework that emphasizes continuous planning, action, monitoring, and community-driven innovation. It calls for dedicated staffing, reliable funding, systematic training, and robust community partnerships to embed cleanliness as a shared culture rather than a one-time activity. If adopted, these recommendations can help bridge the gap between policy intent and on-ground realities, paving the way for HWCs that are not only clean and safe but also trusted and embraced by the communities they serve.

## Figures and Tables

**Figure 6:**
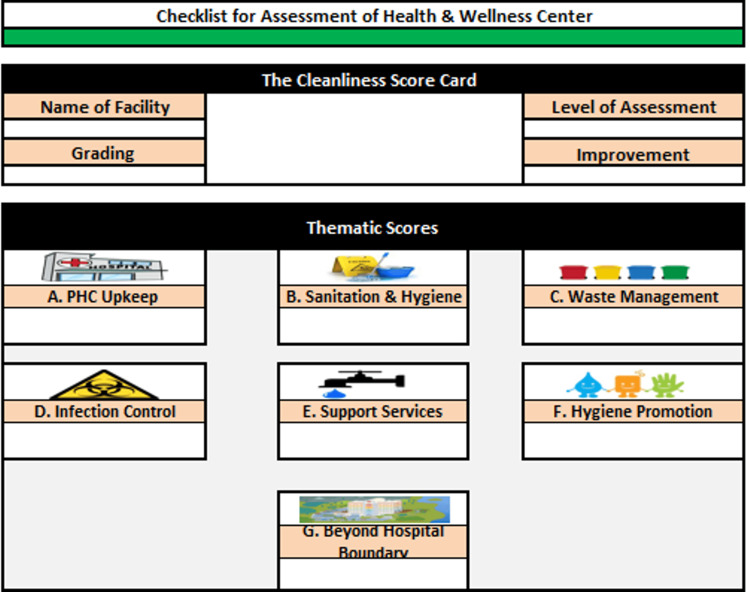
Kayakalp cleanliness scorecard and thematic scores

**Figure 7:**
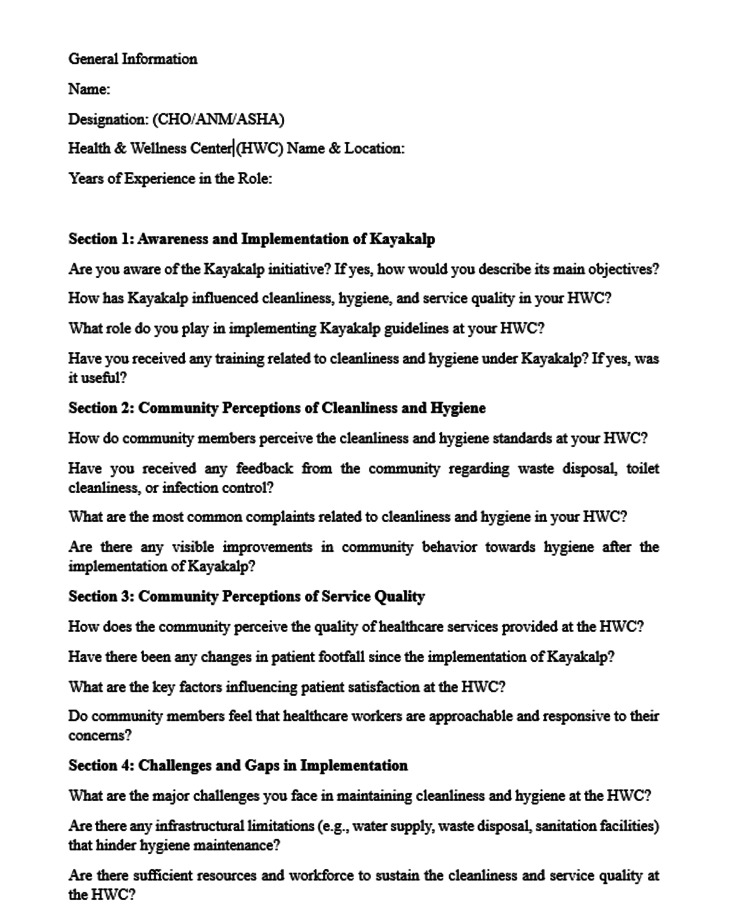
Interview guideline Page 1

**Figure 8:**
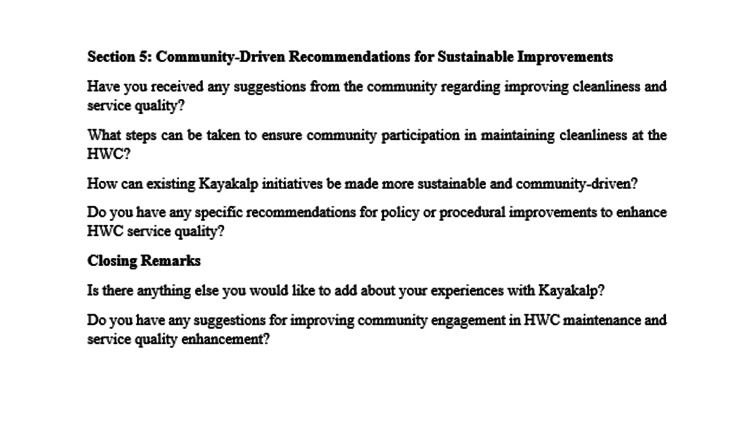
Interview guideline Page 2

## Appendices

Appendix A: Kayakalp facility assessment checklist developed by the Ministry of Health and Family Welfare

**Table 2:** Kayakalp assessment checklist

Ref. No.	Criteria		Assessment Method	Means of Verification			Compliance	Remarks
A.	Centre/Sub Centre level Health and Wellness Centre Upkeep							
A1	Pest & Animal Control							
A1.1	No stray animals within the facility premises		OB/SI	Observe for the presence of stray animals such as dogs, cats, cattle, pigs, etc. within the premises. Also discuss with the facility staff. Also look at the breach, if any, in the boundary wall and presence of secured gate.				
A1.2	Pest Control Measures are implemented in the facility		SI/RR/OB	Check for the evidence at the facility ( Presence of Pests ,Record of Purchase/availability of Pesticides and availability of the rat trap) and interview the staff.				
A2	Landscaping, Gardening & Yoga							
A2.1	Surrounding area/ Open spaces are well maintained		OB	Check that wild vegetation does not exist. Shrubs and Trees are well maintained. Over grown branches of plants/ tree have been trimmed regularly. Dry leaves and green waste are removed.				
A2.2	Provision of Yoga room		OB	Check for adequate space and cleanliness.				
A3	Maintenance of Open Areas							
A3.1	Approach walkway from gate to the facility is even and clean		OB	Check that walkway is even and non-slippery and well maintained				
A3.2	No water logging in open areas		OB	Check for water accumulation in open areas because of faulty drainage, leakage from the pipes, etc.				
A4	Hospital/Facility –Appearance							
A4.1	Walls are well-plastered ,painted and name of the facility is displayed		OB	Check that wall plaster is not chipped-off and the building is painted with yellow color wall & Brown color windows. The paint has not faded away. Name of the Centre is prominently displayed.				
A4.2	Branding of Health & Wellness Centre has been under taken as per current guideline.		OB	Check for:- 1- Outer surface of the building is yellow with specified shade. 2- Windows & their frame in the brown specified shade. 3- Six illustrations drawn on the façade. 4- Logo of NHM and Ayushman Bharat.				
A5	Infrastructure Maintenance							
A5.1	Facility Infrastructure is well maintained		OB	No major cracks, seepage, chipped plaster & floors in the Centre .Periodic Maintenance is done.				
A5.2	Centre has intact boundary wall/Fencing and functional gates at entry		OB	Check that there is a proper boundary wall/fencing of adequate height without any breach.				
A6	Illumination							
A6.1	Adequate illumination in inside and outside of the Centre		OB	Check for Adequate lighting arrangements through natural light or electric bulbs(CFL/LED) inside Centre . Check that Centre front, entry gate and access road are well illuminated.				
A6.2	Use of energy efficient bulbs		OB	Check thatCentre uses energy efficient bulb like CFL or LED for lighting purpose within the Centre Premises				
A7	Maintenance of Furniture & Fixture							
A7.1	Window and doors are maintained		OB	Check, if Window panes are intact, and provided with Grill/ Wire Meshwork. Doors are intact and painted /varnished.				
A7.2	furniture and fixtures are in good condition.		OB	Check that Examination table, foot Step, Table, Chair, stool, etc. are not rusted and are painted. Mattresses are clean and not torn Almirah, Fan , Tube lights etc. are well maintained( As applicable)				
A8	Removal of Junk Material							
A8.1	No junk material within centre premises		OB	Check if unused/ condemned articles, and outdated records are kept in the haphazard manner.				
A8.2	Centre has system for removing junk materials		OB/SI	Check for any system of removing junk from Centre with support from PHC				
A9	Water Conservation							
A9.1	Water supply system is maintained in the Centre		OB	Check for leaking taps, pipes, over-flowing tanks and dysfunctional cisterns. Over-head tank is covered.				
A9.2	Check if the facility has rain-water harvesting system		SI/OB	Check for its functionality and storage system				
A10	Work Place Management							
A10.1	The Staff periodically sorts useful and unnecessary articles at work station		SI/OB	Ask the Staff, how frequently they sort and remove unnecessary articles from their work place.. Check for presence of unnecessary articles.				
A10.2	The Staff arranges the useful articles, records in systematic manner and label them		SI/OB	Check if drugs, instruments, records are not lying in haphazard manner and kept near to point of use in systematic manner. The place has been demarcated for keeping different articles Check that drugs, instruments, records, etc. are labelled for facilitating easy identification.				
B	Sanitation & Hygiene							
B1	Cleanliness of Circulation Area (Corridors, Patient Waiting area)							
B1.1	No dirt,grease,stains, cobwebs, bird nest, dust, vegetation on walls and roof in the circulation area		OB	Check that floors and walls of Corridors, Waiting area etc for any visible or tangible dirt, grease, stains, etc. Check that roof, walls, corners of Corridors, Waiting area for any Cobweb, Bird Nest, etc.				
B1.2	Corridors are cleaned at least once in the day with wet mop		SI/OB	Ask the staff about frequency of cleaning in a day.				
B2	Cleanliness of Clinic room							
B2.1	No dirt,grease,stains, cobwebs, bird nest, dust, vegetation on walls and roof in the Clinic room		OB	Check floors and walls of the clinic room for any visible or tangible dirt, grease, stains, etc. Check that roof, walls, corners of clinic for any Cobweb, Bird Nest, vegetation, etc.				
B2.2	Clinic room is cleaned at least once in a day with wet mop		OB/SI	Ask staff about frequency of cleaning in a day.				
B3	Cleanliness of Procedure Areas(Laboratory / Diagnostic)							
B3.1	No dirt,grease,stains, cobwebs, bird nest, dust, vegetation on walls and roof in the procedure area		OB	Check that floors and walls of storage for any visible or tangible dirt, grease, stains, etc. Check roof, walls, corners of these area for any cobweb, bird nest, vegetation, etc.				
B3.2	Procedure area are cleaned at least once in a day and as required		OB/SI	Ask staff about frequency of cleaning in a day				
B4	Cleanliness of Storage Space							
B4.1	No dirt, grease, stains, cobwebs, bird nest, dust, vegetation on walls and roof in the storage space.		OB	Check that floors and walls of storage for any visible or tangible dirt, grease, stains, etc. Check roof, walls, corners of these area for any cobweb, bird nest, vegetation, etc.				
B4.2	Storage space are cleaned at least once in the day with wet mop		OB/SI	Ask staff about frequency of cleaning in a day				
B5	Cleanliness of Roof top							
B5.1	No dirt, cobwebs, bird nest, junk articles on roof top		OB	Check roof top of the Centre for any dirt, Cobweb, Bird Nest, etc. Check for any junk articles on roof top				
B5.2	Roof top are cleaned at least once in the month		SI/OB	Ask staff about frequency of cleaning				
B6	Cleanliness of Toilets							
B6.1	No dirt/Grease/Stains/ Garbage in Toilets		OB	Check the toilets randomly for any visible dirt, grease, stains, water accumulation in toilets Check for any foul smell in the Toilets				
B6.2	Separate male & female toilets have running water and functional cistern		OB/SI	Check availability of separate male and female toilets Ask staff to operate cistern and water taps				
B7	Use of standards materials and Equipment for Cleaning							
B7.1	Availability of Detergent Disinfectant solution / Hospital Grade Phenyl for Cleaning purpose		SI/OB/RR	Check for good quality cleaning solution preferably a ISI mark. Composition and concentration of solution is written on label. Check with staff if they are getting adequate supply. Verify the consumption records. Check, if the cleaning staff is aware of correct concentration and dilution method for preparing cleaning solution.				
B7.2	Availability of Cleaning Equipment		SI/OB	Check the availability of mops, brooms, collection buckets etc. as per requirement.				
B8	Use of Standard Methods for Cleaning							
B8.1	Use of Two bucket system for cleaning		SI/OB	Check if cleaning staff uses two bucket system for cleaning. One bucket for Cleaning solution, second for wringing the mop. Ask the cleaning staff about the process, Disinfection and washing of mops after every cleaning cycle				
B8.2	Use unidirectional method and out word mopping		SI/OB	Ask cleaning staff to demonstrate the how they apply mop on floors. It should be in one direction without returning to the starting point. The mop should move from inner area to outer area of the room. .				
B9	Monitoring of Cleanliness Activities							
B9.1	Monitoring of cleanliness by Mid Level health provider (MLHP) on daily basis		OB/RR	Ask Mid Level health provider (MLHP) about monitoring mechanism of cleanliness. Check for records				
B9.2	Periodic Monitoring of Housekeeping activities		SI/RR	Periodic Monitoring is done by Block Nodal office at least once in a month.				
B10.	Drainage and Sewage Management							
B10.1	Availability of drainage and sewage system		OB/SI	Centre has a functional septic tank and soak pit within the premises.				
B10.2	No blocked/ over-flowing drains in the facility		OB/SI	Observe that the drains are not overflowing or blocked and they are covered.				
C	Waste Management							
C1	Segregation of Biomedical Waste							
C1.1	Segregation of BMW is done as per BMW management rule,2016 & amendment		OB/SI	General & Biomedical Waste are not mixed together. Display of work instructions for segregation and handling of Biomedical waste				
C1.2	Check if the staff is aware of segregation protocols		SI	Ask staff about the segregation protocol (Red bag for re-cyclable, Glassware into puncture proof and leak proof boxes and container with blue marking, etc.)				
C2	Collection and Transportation of Biomedical Waste							
C2.1	Centre waste is collected and transported in safe manner		OB	Check for records of linkage with CWTF operator or has functional deep burial pits within the facility.				
C2.2	The waste is transported in closed bag		OB	Check availability of bag for transportation of waste.				
C3	Sharp Management							
C3.1	Disinfection of Broken / Discarded Glassware is done as per recommended procedure		OB/SI/ RR	Check if such waste is either pre-treated with 1% to 2% Sodium Hypochlorite (having 30% residual chlorine) for 20 minutes				
C3.2	Sharp Waste is stored in Puncture proof containers		OB/SI	Check availability of Puncture & leak proof container (White Translucent) at point of use for storing needles, syringes with fixed needles, needles etc.				
C4	Storage of Biomedical Waste							
C4.1	BMW should not be stored more than recommended time		OB	Check if facility has functional Deep burial and sharp pit, they should dispose BMW on daily basis/ Facility having linkage with CTF should not store BMW more than 48 hours				
C4.2	Facility for storage of BMW		SI/RR	Facility with deep burial and sharp pit not required any storage facility/ Facility with linkage to CTF requires an isolated place with separate bins for storage of BMW				
C5	Disposal of Biomedical waste							
C5.1	Centre has adequate facility for disposal of Biomedical waste		RR/OB/SI	The Health facility within 75 KM of CTF shall have a valid contract with a Common Treatment facility for disposal of Bio medical waste. Or else facility should have Deep Burial Pit and Sharp Pit within premises of Health facility. Such deep burial pit should have approval of the Prescribed Authority and would meet the norms.				
C5.2	Facility manages recyclable waste as per approved procedure		OB/SI	Check management of IV Bottles (Plastic), Syringes, etc. (shredding/mutilation or a combination of sterilization and shredding and handed over to registered vendor are ensured after linkage with block PHC/CHC).				
C6	Management Hazardous Waste							
C6.1	Staff is aware of Mercury Spill management		SI/OB	Ask staff what he/she would do in case of Mercury spill. (If facility is mercury free, give full compliance)				
C6.2	Availability of Mercury Spill Management Kit		SI	Check availability of Mercury Spill Management Kit.(If facility is mercury free, give full compliance)				
C7	Solid General Waste Management							
C7.1	Disposal of General Waste		OB/SI	There is a mechanism of removal of general waste from the facility and its disposal.				
C7.2	Innovations in managing general waste		OB/SI/ RR	Look for efforts of the health facility in managing General Waste, such as Recycling of paper waste, vermicomposting, waste to energy initiative, etc.				
C8	Liquid Waste Management							
C8.1	Facility has provision of liquid waste management		OB/SI/ RR	Check for onsite provision of liquid waste disinfection set-up				
C8.2	Liquid waste is made safe before mixing with other waste water		OB/SI	Check for the procedure - staff interview and direct observation				
C9	Equipment and Supplies for Bio Medical Waste Management							
C9.1	Availability of Bins and plastic bags for segregation of waste at point of use		OB/SI	One set of appropriate size bins at each point of generation for Biomedical and General waste. Check all the bins are provided with chlorine free plastic bags. Ask staff about adequacy of supply.				
C9.2	Availability of Needle/ Hub cutter and puncture proof boxes		OB/SI	At each point of generation of sharp waste				
C10	Statuary Compliances							
C10.1	Centre has a valid authorization for Bio Medical waste Management from the prescribed authority		RR	Check for the validity of authorization certificate				
C10.2	Centre maintains records, as required under the Biomedical Waste Rules 2016 and Amendment		RR	Check following records - a. Annual report submission b. Yearly Health Check-up record of all handlers c. BMW training records of all staff (once in year training) d. Immunisation records				
D	Infection Control							
D1	Hand Hygiene							
D1.1	Availability of Sink and running water at point of use		OB	Check for washbasin with functional tap, soap and running water at all points of use				
D1.2	Staff is adheres to hand washing protocol		SI	Check Display of Hand washing Instructions Ask facility staff to demonstrate 6 steps of normal hand wash and 5 moments of hand washing				
D2	Personal Protective Equipment (PPE)							
D2.1	Use of Gloves during procedures and examination		SI/OB	Check, if the staff uses gloves during examination, and while conducting procedures				
D2.2	Use of Masks, gloves and aprons		SI/OB	Check, if staff uses mask, gloves, aprons as applicable				
D3	Personal Protective Practices							
D3.1	The staff is aware of use of gloves, when to use (occasion) and its type		SI/OB	Check with the staff when do they wear gloves, and when gloves are not required. The Staff should also know difference between clean & sterilized gloves and when to use				
D3.2	No re-use of disposable personal protective equipment		SI/OB	Check that disposable gloves and mask are not re-used. Reusable Gloves and mask are used after adequate sterilization.				
D4	Decontamination and Cleaning of Instruments							
D4.1	Staff knows how to make Chlorine solution		SI	Ask the staff about the procedure of making chlorine solution and its frequency				
D4.2	Decontamination of instruments and Surfaces like examination table, dressing tables etc.		SI/OB	Check whether instruments are decontaminated with 0.5% chlorine solution for 10 minutes. Check instruments are cleaned thoroughly with water and soap before sterilization Ask staff when and how they clean the surfaces either by chlorine solution or Disinfectant like carbolic acid				
D5	Reprocessing of reusable instruments and equipment							
D5.1	Adherence to Protocols for items that come in contact with intact skin		SI/OB/RR	Check reusable instruments like thermometer, Stethoscope etc. are free from visible contamination and they are washed with soap and water before use.				
D5.2	Adherence to Protocol for High Level disinfection		SI/OB	Check with the staff process about of High Level disinfection using Boiling for 20 minutes with lid on, soaking in 2% Glutaraldehyde/Chlorine solution for 20 minutes.				
D6	Spill Management							
D6.1	Staff is aware of how to manage spills		SI	Check for adherence to protocols				
D6.2	Spill management protocols are displayed at points if use		SI/OB	Check for display				
D7	Isolation and Barrier Nursing							
D7.1	Infectious patients are separated from other patients		OB/SI	Check patients with respiratory infectious cases are separated from general patients in clinic room.				
D7.2	Staff is aware about Standard Precautions		OB	Ask staff about Standard precautions and how they adhere to it.				
D8	Infection Control Program							
D8.1	Monitoring of infection control practices		RR/SI	Check if the Centre has a system to monitor infection control practices by Mid-level Health provider (MLHP) ,Village Health Sanitation and Nutrition Committee at least in a month.				
D8.2	Immunization and medical check-up of Service Providers		RR/SI	Check record of staff, immunized against Hepatitis B and at least once a year medical check-up done.				
D9	Surveillance Activity							
D9.1	Surveillance activity at the centre		RR/SI	Check for surveillance about any abnormal increase in cases of diarrhea /dysentery, fever with rigors, fever with rash, fever with jaundice or fever with unconsciousness and early reporting to concerned PHC as per IDSP guidelines.				
D9.2	Facility reports all notifiable diseases and events		RR/SI	Check facility has list of all notifiable disease needs immediate/periodic reporting to higher authority. Check records that notifiable disease have been reported in program such as IDSP and AEFI Surveillance.				
D10	Environment Control							
D10.1	Cross-ventilation		OB/SI	Check availability of Fans/ air conditioning/ Heating/ exhaust/ Ventilators as per environment condition and requirement				
D10.2	Preventive measures for air borne infections has been taken		OB/SI	Check location of the Centre , it should be away from Garbage dump Cattle shed, Stagnant pool, Pollution from industry				
E	SUPPORT SERVICES							
E1	Laundry Services & Linen Management							
E1.1	Available linens are clean		RR/SI	Check linen such as table cloth, bedsheets, curtains etc. are clean and spotless				
E1.2	Arrangements for washing linens		OB/SI	Check facility has in-house or outsourced arrangements for washing linens at least once in a week.				
E2	Water Sanitation							
E2.1	The facility receives adequate quantity of water as per requirement		RR/SI/PI	Water is available on 24x7 basis at all points of usage				
E2.2	There is storage tank for the water and tank is cleaned periodically		RR	The Centre have capacity to store 48 hours water requirement Water tank is cleaned at six monthly interval and records are maintained.				
E3	Storage Space							
E3.1	Medicines are arranged systematically		OB/SI	Check all the shelves/racks containing medicines are labelled in Storage and drug store. Heavy items are stored at lower shelves/racks Fragile items are not stored at the edges of the shelves Drugs and consumables are stored away from water and sources of heat, direct sunlight etc. Drugs are not stored at floor and adjacent to wall				
E3.2	Cold storage equipment's are clean and managed properly		OB	Check refrigerators / Ice packs are clean Check if there is a practice of regular cleaning. Cold box are not been used for purpose other than storing drugs and vaccines.				
E4	Housekeeping services							
	Routine Cleaning of the facility at least once in a day		OB/RR	Cleaning includes dry and wet mopping				
E4.2	Thorough Cleaning of the facility fortnightly		SI/RR	Thorough cleaning with warm water and soap/detergent				
E5	Outreach Services							
E5.1	Biomedical waste generated during outreach session are transported to the centre on the same day		RR/SI	Check the records and ask staff				
E5.2	Reporting PHC monitor cleanliness and hygiene of outreach sessions and the center		RR/ SI	Check records that reporting PHC staff/In charge has checked cleanliness and hygiene of the centre and its outreach session at least monthly basis.				
F	Hygiene Promotion							
F1	Community Monitoring & Patient Participation							
F1.1	Patients are made aware of their responsibility of keeping the health facility clean		PI/OB	Ask patients about their roles & responsibilities with regards to cleanliness. Patient’s responsibilities should be prominently displayed				
F1.2	Patient rights and responsibility are displayed		SI/RR/OB	Check for IEC regarding the role and responsibility				
F2	Information Education and Communication							
F2.1	IEC regarding importance of Hygiene practices are displayed		OB	Check IEC regarding hand washing, water sanitation, use of toilets are displayed in local language				
F2.2	IEC regarding Swachhta Abhiyan is displayed within the facilities’ premises		OB	Should be displayed prominently in local language				
F3	Leadership and Team work							
F3.1	Staff worked as a team to improve sanitation and hygiene of the facility		SI/OB	Ask staff about sanitation and hygiene				
F3.2	Roles and responsibility of different staff members have been assigned and communicated		SI/RR	Ask different members about their roles and responsibilities to make facility clean				
F4	Training and Capacity Building and Standardization							
F4.1	Bio medical waste Management training has been provided to the staff		SI/RR	Ask staff and look for any record				
F4.2	Infection control Training has been provided to the staff		SI/RR	Check staff are trained at the time of induction and at least once in every year				
F5	Staff Hygiene and Dress Code							
F5.1	Centre has dress code policy for all cadre of staff		OB/SI	Mid-Level Healthcare Provider and other health workers are in dress code. Check for I card and name plates				
F5.2	There is a regular monitoring of hygiene of staff		SI/OB	Check about personal hygiene and clean dress of staff				
G	Beyond Hospital Boundary							
G1	Promotion of Swachhata & Coordination with Local bodies							
G1.1	Facility is situated in ODF area		RR/SI	Centre is located in ODF village				
G1.2	Local community actively participates in VISHWAS campaign		RR/SI	Check for activities carried out under the leadership of VHSNCs for improving water, sanitation and hygiene situation during VISHWAS campaign.				
G1.3	Implementation of IEC activities related to ' Swachh Bharat Abhiyan'		OB/RR/SI	Check for any pamphlets/Posters/wall writing-promoting use of toilets, hand washing, safe drinking water and tree plantation etc.				
G1.4	The Facility coordinates with local Gram Panchayat and NGOs for improving the sanitation and hygiene		RR/SI	Look for evidence of collective action for improving water, sanitation and hygiene.				
G1.5	The Facility coordinates with other departments for improving Swachhata		RR/SI	Look for evidence of coordination with departments such as Education (school programs on hygiene promotions), Water and Sanitation (making area ODF), PWD (Repair & Maintenance), Forest Department (Plantation Drive) etc. which contributes strengthening towards of hygiene & sanitation				
G2	Cleanliness of approach road and surrounding area							
G2.1	Area around the facility is clean, neat & tidy		OB	Check for any litter/garbage/refuse and water logging in the surrounding area of the facility.				
G2.2	On the way signages are available		OB/SI	Check for directional signage with name of the facility on the approach road.				
G2.3	Approach road is clean and even		OB/SI	Check that approach road are clean and even				
G2.4	All drain and sewer are covered.		OB	Check for overflowing drains surrounding the facility.				
G2.5	Functional street lights are available along the approach road		OB/SI	Check for street lights and their functionality.				
G3	Aesthetics and amenities of Surrounding area							
G3.1	Parks and green areas of surrounding area are well maintained		OB/SI	Check that there no wild vegetation & growth in the surroundings. Shrubs and trees are well maintained. Dry leaves and green waste are removed regularly.				
G3.2	No unwanted/broken/ torn / loose hanging posters/ billboards.		OB	Check that facility surrounding are not studded with irrelevant and out dated posters, slogans, wall writings, graffiti, etc.				
G3.3	No loose hanging wires in and around the bill boards, electrical poles, etc.		OB	Check for any loose hanging wires.				
G3.4	Availability of public toilets in surrounding area		OB/SI	Check for separate toilets for male and female and they are conveniently located and clean.				
G3.5	Availability of adequate parking stand in surrounding area		OB/SI	Check for parking stand for auto/ rickshaw/taxi etc., and they are not parked haphazardly.				
G4	Maintenance of surrounding area and Waste Management							
G4.1	Availability of bins for General recyclable and biodegradable wastes		OB	Check availability adequate number of bins for Biodegradable and recyclable general waste in the nearby area.				
G4.2	Availability of garbage storage area		OB	Garbage storage area is away from residential/commercial areas and is covered/fenced. It is not causing public nuisance.				
G4.3	Innovations in managing waste		OB/SI	Check, if certain innovative practices have been introduced for managing general waste e.g. Vermicomposting, Re-cycling of papers, Waste to energy, Compost Activators, etc.				
G4.4	Surrounding areas are well maintained		OB	Check that there is no over grown shrubs, weeds, grass, potholes, bumps etc. in surrounding areas				
G4.5	Regular repairs and maintained of roads		OB/SI/RR	Check current condition of the road				

Appendix B: interview guide
